# Bee wisdom: exploring bee control strategies for food microflora by comparing the physicochemical characteristics and microbial composition of beebread

**DOI:** 10.1128/spectrum.01818-23

**Published:** 2023-10-06

**Authors:** Ying Wang, Lanting Ma, Baohua Xu

**Affiliations:** 1 College of Animal Science and Technology, Shandong Agricultural University, Tai’an, China; Fujian Agriculture and Forestry University, Fuzhou City, Fujian, China

**Keywords:** ecosystem, beebread, physicochemical characteristics, microflora

## Abstract

Beebread serves not only as a vital food source for bees but also as a significant reservoir of microbes for their intestinal tracts. The distribution of beebread in honeycomb cells is characterized by distinct vertical patterns; however, the vertical distribution patterns of the physicochemical characteristics and microbial composition of beebread remain unclear. In this study, we collected beebread samples from nine colonies in three representative ecosystems (forest, forest-urban, and urban-farmland ecosystems) and systematically assessed the physicochemical characteristics and bacterial diversity in three beebread layers (lower, middle, and upper). We found that upper beebread samples had lower pH; higher proportions of fructose and sucrose; and lower proportions of total solids, crude protein, and ash compared to lower samples. Moreover, 16S rDNA sequencing showed that the dominant bacterial phyla and genera were relatively simple and highly conserved across the three ecosystems. Overall, the microbial community richness of upper beebread was significantly higher than that of lower beebread, and Bacteroidetes and Acidobacteria were significantly decreased in lower compared to upper beebread. Meanwhile, the relative abundance of dominant taxa in the same beebread layer differed by ecosystem types. These findings suggest that bees employ fermentation processes to selectively enrich microbes present in flower pollen, leading to homogenous and relatively stable microbial diversity in beebread. Our study expands on the current understanding of beebread and highlights the significance of fermentation in its production. Furthermore, our findings provide a useful reference for further in-depth exploration of the interactions between food, food microbes, and bee gut microbes.

**IMPORTANCE:**

Bees are a valuable model for investigating the relationship between environmental factors, gut microbiota, and organismal health. Beebread, produced from collected pollen, is a natural food source and a primary reservoir of gut microorganisms. Although pollen typically has diverse bacterial species, beebread has low species richness and bacterial abundance. Consequently, considerable attention has been paid to the adaptive strategies employed by honey bees to cope with the microorganisms within their food environment during co-evolution with plants. This study identified the distribution patterns of beebread's physicochemical characteristics, showing how bees use fermentation to enrich specific microbes. These findings help understand the relationship between environmental and food-associated microbes and bee intestinal microbiota. They also bridge gaps in the literature and provide a valuable reference for studying the complex interplay between these factors.

## INTRODUCTION

Honeybees are important pollinators and play a key role in global crop production and ecosystem maintenance. However, in recent years, honeybee populations have declined dramatically worldwide, and their nutrition and health are of great concern to scientists (1). The honeybee gut is inhabited by a large number of microbes, which play a very important role in ensuring the health of honeybees by providing them with nutrients (2, 3) and defending them against pathogenic bacteria (3 – 5). The microbes in honeybees are relatively simple and highly conserved (6 – 8), and the core microbial species usually consist of only five to nine species such as *Lactobacillus Firm-4*, *Lactobacillus Firm-5*, *Snodgrassella alvi*, and *Gilliamella apicola* (8, 9). Newborn honeybees have almost no microbes in their gut (8); they feed heavily on beebread and honey and establish a relatively stable gut microbial community around 9 days of age (10 – 12). It is important to study the colonization pattern of these gut microbes in order to maintain bee health.

Food is the main source of microbes for the bee’s gut. Not all the microbes in the bee’s diet are useful to the bee, so the bee selects for those it needs—by fermentation. Honey and beebread are the main food items for honeybees, and they are both made by bees collecting nectar or pollen from plants and then processing it. The process of making beebread is more complex than that of honey, and it varies between different bee species (5). Plant pollen is collected by bees, mixed with glandular secretions (including enzymes), loaded into hive cells, and covered with honey (13). Under anaerobic conditions, combined with the appropriate temperature and humidity in the colony, the microbes in the pollen and the enzymes secreted by the bees work together to produce beebread (13). Well-made beebread is rich in proteins (15–28%) including enzymes, sugars (24–35%), lipids (3–9%), vitamins and minerals (3–5%), and other nutrients (5, 14, 15) and is more easily digested and absorbed by bees than pollen. Beebread is also a “microbial reservoir” for bees, containing almost all the species of intestinal microbes of bees (5, 16, 17). Therefore, studying the microbial communities in beebread will expand our understanding of the honeybee gut microbial communities.

If the nutrient composition or microbes in beebread in the hive vary, the honeybee gut microbes may become unstable. The honeybee’s hive is hexagonal and cylindrical, 12.1–16.9 mm deep, and closed at the bottom and open at the top (18). The unique structure of the hive provides good conditions for fermentation. When the hive is almost filled with pollen, the bees will apply a thin layer of honey (19) to insulate the pollen. The addition of more honey to mature beebread makes a noticeable difference in the appearance of the upper layer compared to the lower layer: the upper layer looks moister and more delicate, while the lower layer appears dry and coarse. In addition, the fermentation process that converts pollen into beebread is accompanied by huge changes in the microbes. Pollen and bee-collected pollen contain a huge number and variety of bacteria (10, 11, 20 – 26), which vary according to the origin of the plants, their geographical location, etc. Pollen and bee-collected pollen often contain large numbers of microbes that are harmful to bees (10, 27 – 29), and if bees consume this contaminated pollen directly, it will undoubtedly pose a significant health risk to them. However, the variety and abundance of microbes in pollen decrease dramatically [<1,000 operational taxonomic units (OTUs)] when it is changed to beebread (26) (Table S1) The gut microbiota of honeybees is susceptible to changes in the honeybees’ food source (5). Similar to the gut microbiota of honeybees, the microbial composition of beebread, although relatively simple (5, 16), may differ by region and food source (5, 16, 23). However, most studies have focused on the overall nutritional value or microbial community of beebread in the hive, and fewer studies have been conducted on different beebread layers. Therefore, further in-depth studies are necessary to explore the vertical distribution patterns of nutrition and microbes in beebread in the hive.

In this study, we collected beebread from colonies in three typical ecosystems (forest, forest-urban, and urban-farmland) and characterized the spatial distribution patterns of physicochemical characteristics (including nutrients) and microbes in the upper, middle, and lower layers of beebread. We sought to test the hypotheses that (i) the physicochemical characteristics (including nutrient composition) differ by beebread layer; (ii) the microbial composition of beebread differs by beebread layer; and (iii) the microbial composition of beebread is influenced by ecosystem type. This study will enrich the understanding of the composition and microbiology of beebread.

## RESULTS

### Physicochemical analysis

**FIG 1:**
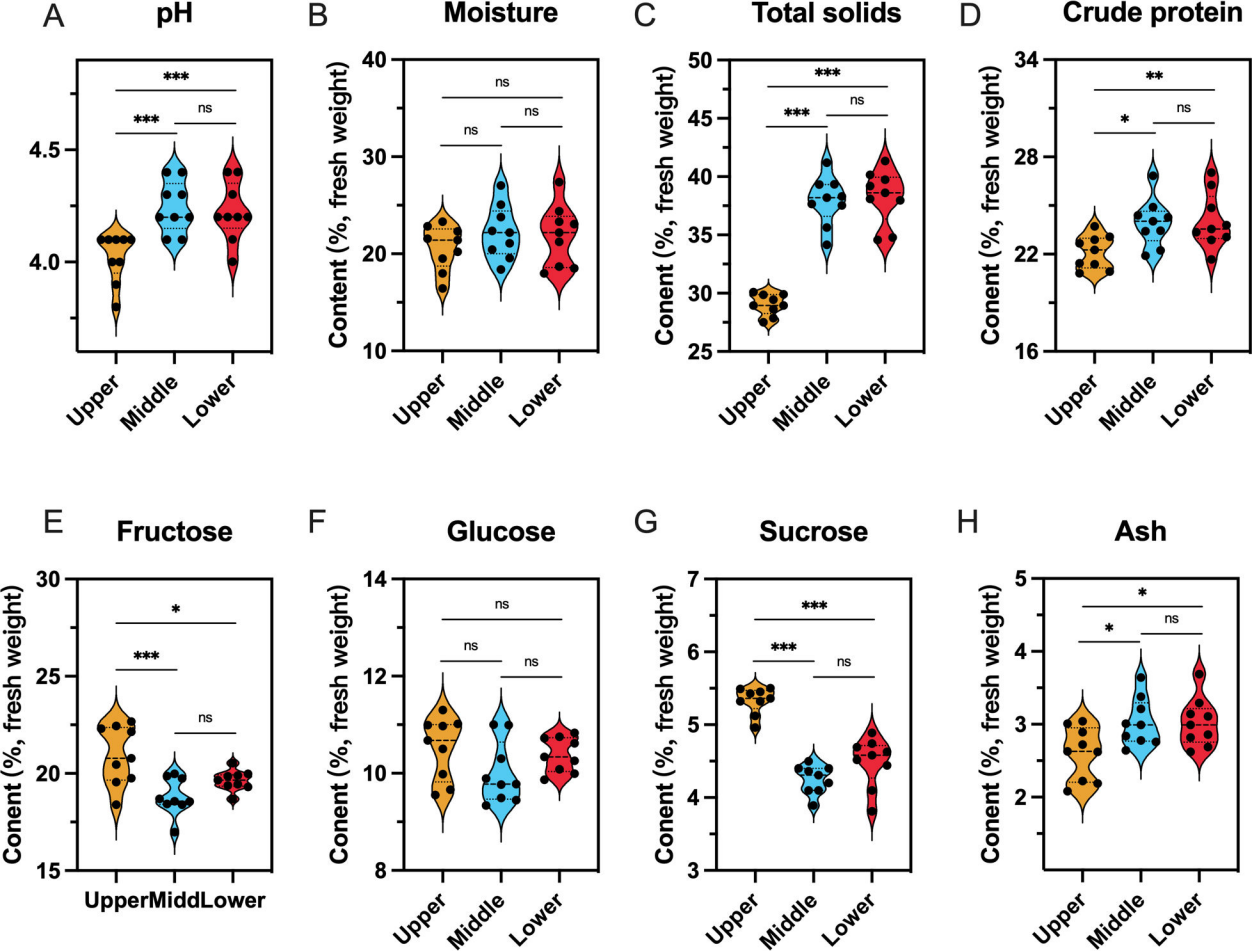
Physicochemical characteristics of beebread samples. *P < 0.05, **P < 0.01 and ***P < 0.001 based on LSD test (*n* = 9). ns, not significant.

Various physicochemical characteristics of beebread (pH, total solids, crude protein, fructose, sucrose, and ash) exhibited large variations among different beebread layers (Fig. 1; Table S2). The characteristics of the upper beebread were completely different from those of the middle and lower beebread, while those of the latter two were similar [least significant difference (LSD) test, all *P* > 0.05]. More precisely, upper beebread had significantly lower pH, total solids, crude protein, and ash, and significantly higher fructose and sucrose than the middle and lower beebread (LSD test, all *P* < 0.05). There were no significant differences in moisture or glucose among the different layers (LSD test, all *P* > 0.05).

### Sequencing output and OTU clustering

Illumina sequencing generated 3,481,505 raw pair-end reads. After quality filtering, overlap assembly, and clustering, 3,376,806 clean tags were obtained. The mitochondrial and plastid sequences and the singletons were removed, leaving 3,009,403 effective tags (Table S3) grouped into 21,209 OTUs, with an average similarity level of 86.45%.

**FIG 2:**
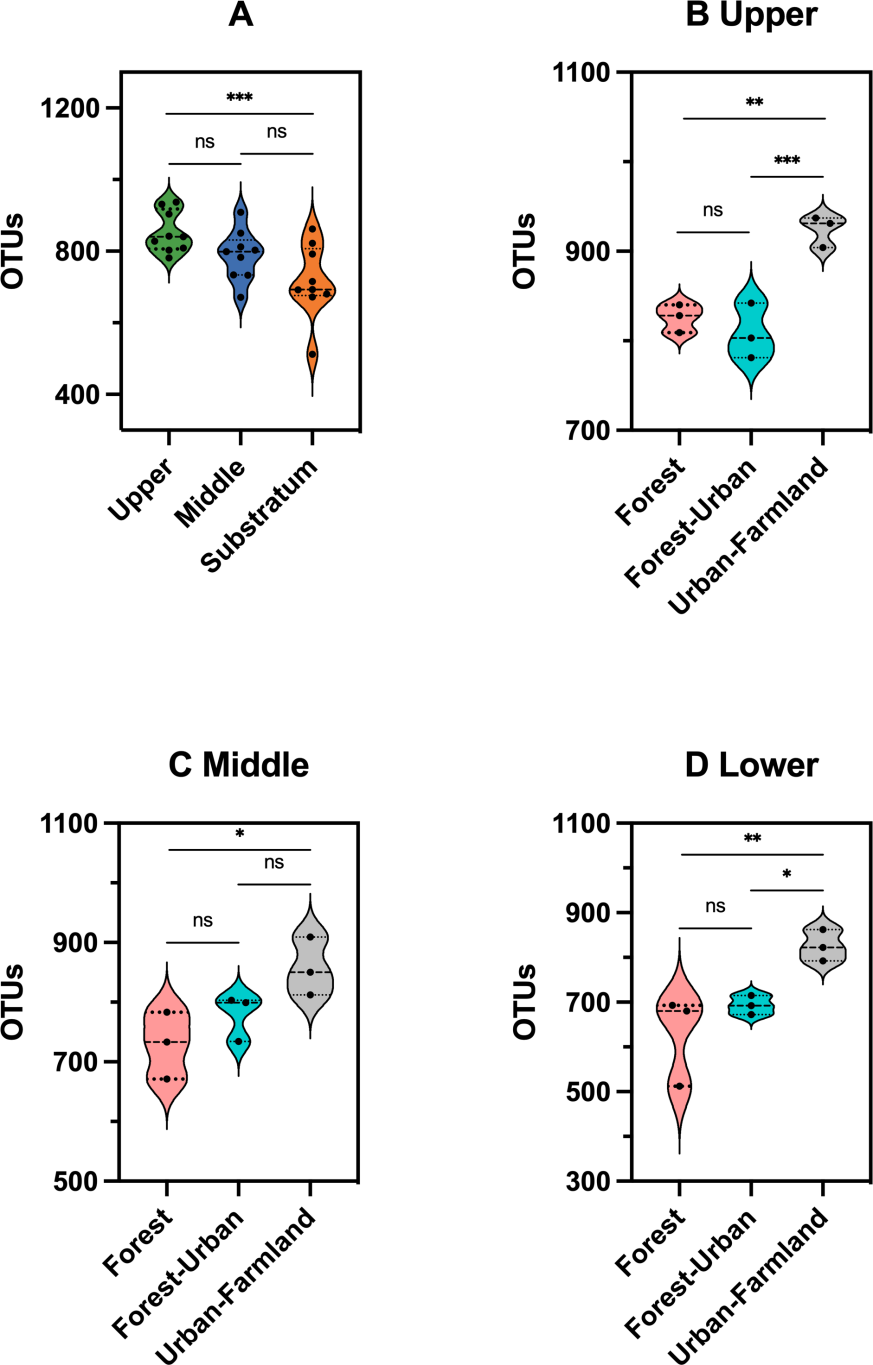
Comparison of OTUs in beebread samples. OTUs in beebread samples of (**A**) different layers (*n* = 9) and (**B**) upper, (**C**) middle, and (**D**) lower layers from different ecosystem types (*n* = 3). *P < 0.05, **P < 0.01, ***P < 0.001 based on LSD test.

The number of OTUs was significantly higher in the upper beebread than the lower beebread (*P* = 0.001; Fig. 2A), but there were no differences between pairs of adjacent layers (all *P* > 0.05). There were significantly more OTUs in upper, middle, and lower beebread from urban-farmland areas compared to forest areas (*P* = 0.002, *P* = 0.018, and *P* = 0.009, respectively; Fig. 2B through D).

### Alpha-diversity and beta-diversity

**FIG 3:**
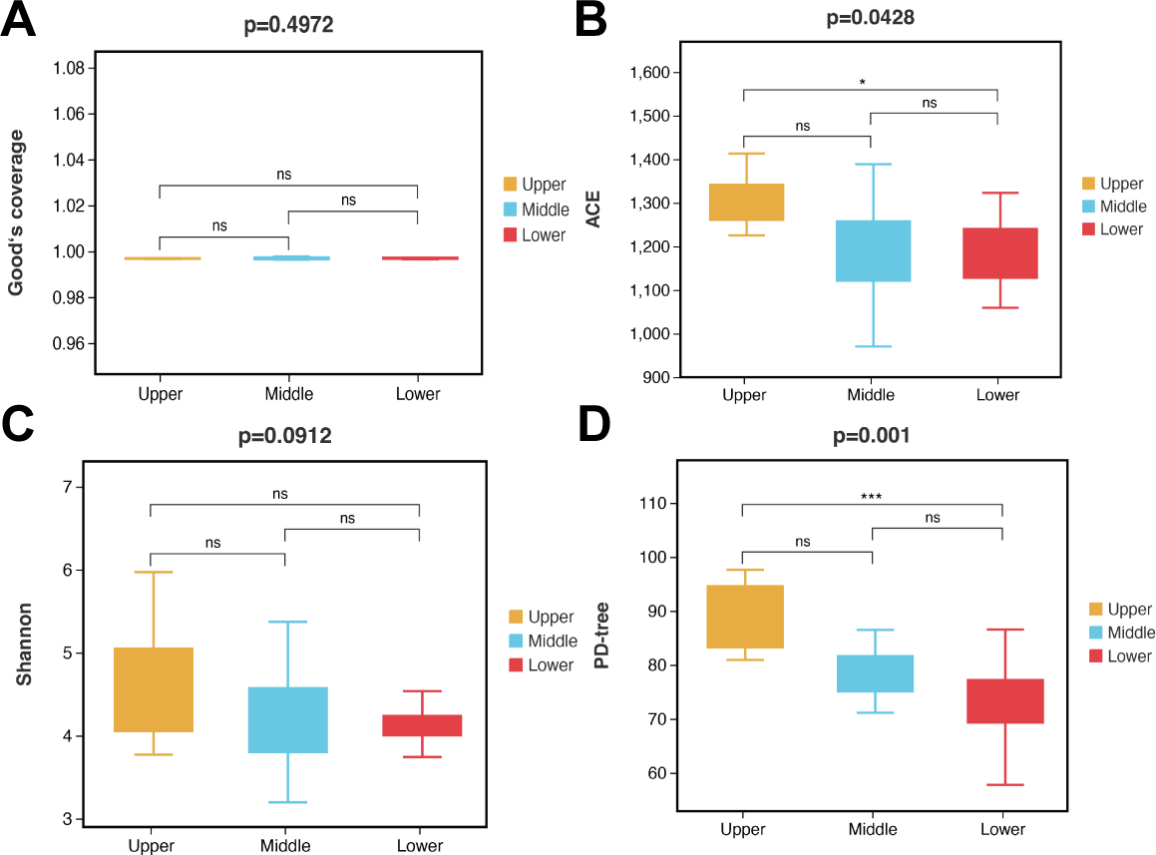
Comparison of alpha-diversity indexes of the microbiota of three beebread layers. Values are mean ± standard deviation. *P < 0.05, ***P < 0.001 based on Tukey’s honestly significant difference test.

**FIG 4:**
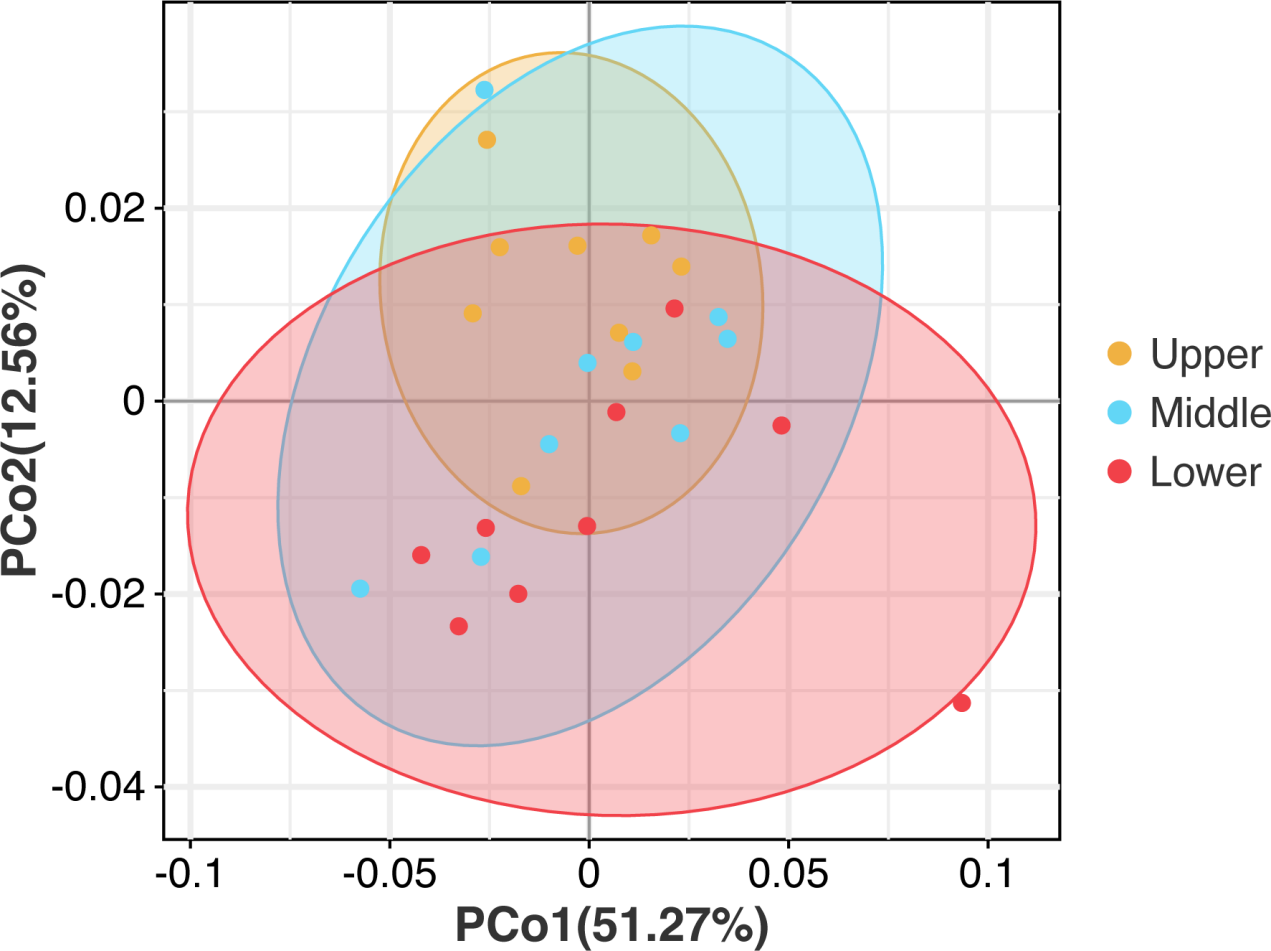
Principal co-ordinate analysis of beebread microbial communities based on weighted UniFrac dissimilarity matrix (*n* = 9 per layer).

To compare the microbial diversity of beebread among beebread layers, we conducted alpha- and beta-diversity analyses. The alpha-diversity analysis involved calculating the abundance-based coverage estimator (abundance-based coverage estimator [ACE] and Chao1; community richness), Shannon index and Simpson index (community diversity), Good’s coverage (sequencing depth), and phylogenetic diversity (PD)-tree index (Table S4). There were no significant differences in the Shannon index or Good’s coverage among beebread layers (Fig. 3A and 4C; Table S4). ACE was significantly higher in upper beebread than lower beebread (Fig. 3B; Table S4). PD-tree index was significantly higher in upper beebread than lower beebread (Fig. 3B; Table S4).

The principal co-ordinate analysis (PCoA) based on weighted UniFrac distances showed large overlaps in the microbiota of upper, middle, and lower beebread, which indicated that the microbial community structure of different layers was similar (Fig. 4). These results were confirmed by Adonis [permutational multivariate analysis of variance ([PERMANOVA)] multivariate comparisons, which showed that there was no significant difference in the microbiota structure of different beebread layers (Table S5).

Alpha- and beta-diversity analyses were also performed for same-layer beebread samples from the three ecosystem types. There were no significant differences in ACE or Shannon indexes among the three ecosystem types for any layer, indicating that the ecosystem types had limited effects on the microbial richness and evenness of beebread (Fig. S1). For the upper and lower layers, the PD-tree index was significantly higher in urban-farmland compared to forest and forest-urban beebread. For the middle layer, the PD-tree index was significantly higher in urban-farmland compared to forest beebread (Fig. S1).

To visualize the clustering of the beebread samples based on their microbial communities, unweighted PCoA was performed. Regarding microbial composition, urban-farmland beebread samples were more centralized and resembled each other compared to the forest and forest-urban beebread samples (Fig. S2). For each layer, there was significant divergence among the forest, forest-urban, and urban-farmland groups based on Adonis (PERMANOVA) analysis (upper layer: *R* = 0.3025, *P* = 0.01; middle layer: *R* = 0.3087, *P* = 0.006; lower layer: *R* ^2^ = 0.3591, *P* = 0.006).

### Alterations of beebread microbiota composition

**FIG 5:**
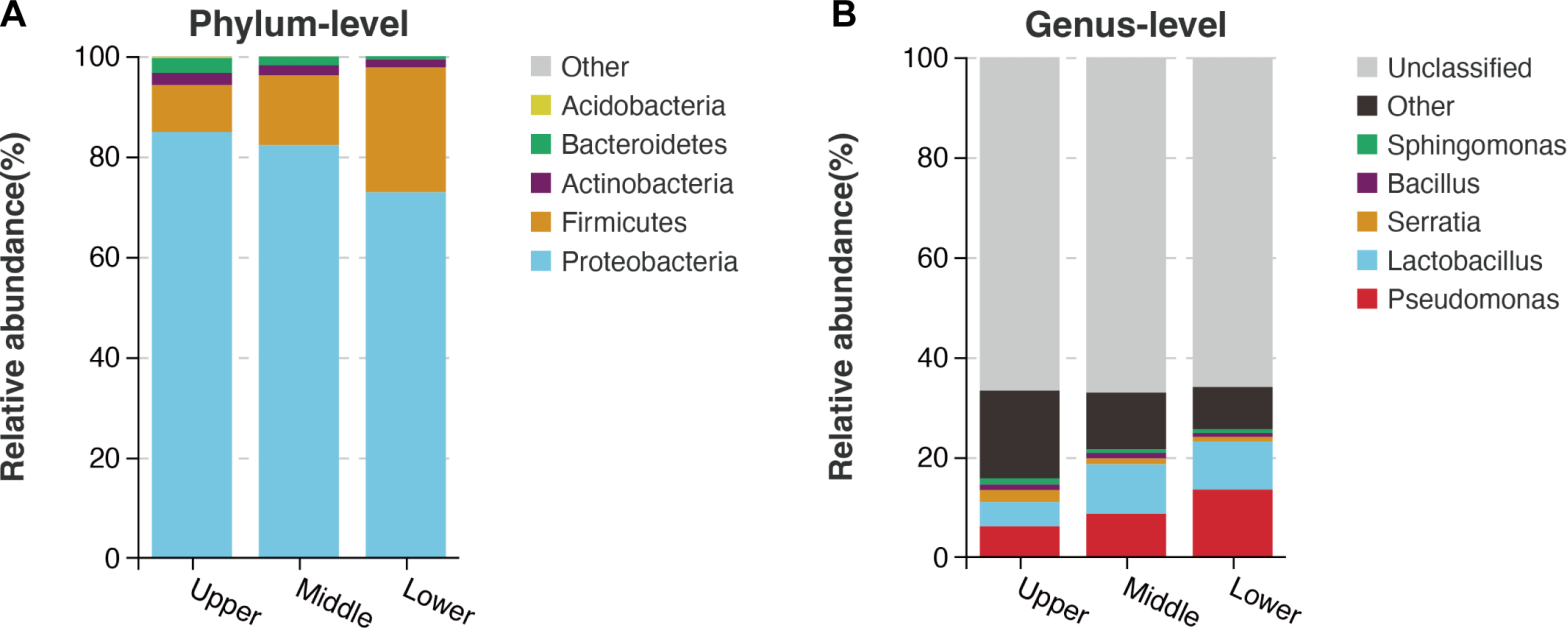
Comparison of major bacterial phyla and genera among the three beebread layers. Relative abundances of the five most abundant (**A**) phyla and (**B**) genera in the three layers.

Based on the species annotation results, the five most abundant bacterial phyla and genera were determined for the upper, middle, and lower beebread, and then histograms were generated to visualize the relative abundances of the bacteria (Fig. 5).

Proteobacteria (72.83–84.87%), Firmicutes (9.33–24.89%), Actinobacteria (1.65–2.45%), Bacteroidetes (0.60–2.93%), and Acidobacteria (0.0039–0.28%) were the five most abundant phyla (Fig. 5A; Table S6). Bacteroidetes and Acidobacteria were significantly decreased in lower compared to upper beebread (Table S6). There were no significant differences in Proteobacteria, Firmicutes, or Actinobacteria among the three layers (all *P* > 0.05; Table S6).

For all layers, *Pseudomonas*, *Lactobacillus*, *Serratia*, *Bacillus*, and *Sphingomonas* were the five most abundant genera (Table S7). There were no significant differences in these five genera among the layers (all *P* > 0.05; Table S7).

Next, we investigated the microbial composition of different beebread layers from different ecosystem types. Proteobacteria, Firmicutes, Bacteroidetes, and Actinobacteria were the four most abundant bacteria in all groups (Fig. S3A through C). Acidobacteria was only detected in upper and middle beebread, and *Gemmatimonadetes* was only detected in lower beebread. There were no significant differences in the relative abundances of the five most dominant phyla in each layer by ecosystem type, except that Actinobacteria was significantly decreased in lower beebread from forest areas compared to forest-urban and urban-farmland areas (Table S8).

At the genus level, the microbial communities in all beebread layers from the three ecosystem types were all dominated by *Pseudomonas*, *Lactobacillus*, and *Serratia*. In addition to these three dominant genera, the other two dominant genera in upper, middle, and lower beebread were *Aeromonas* and *Sphingomonas*, *Bacillus* and *Curvibacter*, and *Bacillus* and *Sphingomonas*, respectively (Fig. S3D through F). Regarding the upper layer, the relative abundance of *Pseudomonas* significantly differed for all ecosystem type comparisons (all *P* < 0.5, Table S9). Additionally, *Aeromonas* was significantly higher in forest beebread than forest-urban and urban-farmland beebread (Table S9). Regarding the middle layer, there were no significant differences in any of the five most dominant genera by ecosystem type (Table S9). Regarding the lower layer, *Pseudomonas* and *Lactobacillus* significantly differed for all ecosystem type comparisons; *Pseudomonas* was the most dominant genus in urban-farmland beebread, and *Lactobacillus* was the most dominant genus in forest-urban beebread. Additionally, *Sphingomonas* was significantly higher in urban-farmland beebread than forest and forest-urban beebread (Table S9).

### Indicator species analysis

To identify whether the beebread microbiomes were highly sensitive to the storage location parameters, an indicator value analysis (indicator value *P* < 0.05) was conducted basing on the microbial community data. In total, 19 bacterial species were identified as significant indicators for beebread depth (Fig. S4). The upper-layer beebread were identified in the bacterial species *Comamonas_aquatica*, *Frischella*_*perrara*, *Bacteroides_sp_PH5-19*, *Gilliamella_apicola*, *Melissococcus_plutoniu*s, *Nocardioides_sp, Prevotella_sp_DJF_CP65*, *Blattella_germanica_German_cockroach*, *Lactobacillus_sp_RA2113*, *Brevundimonas_sp_218PP*, *Geobacter_sp*, *Bacillus_gibsonii*, *Streptococcus_constellatus_subsp_constellatus*, *Pseudoxanthobacter_liyangensis*, *Paenibacillus_nicotianae*, *Lactobacillus_rhamnosus*, *Pleomorphomonas_oryzae_DSM_16300*, *Azorhizobium_sp_F1* and *Cloacibacterium_normanense*. *Nocardioides_sp*, *Lactobacillus_rhamnosus*, *Streptococcus_constellatus_subsp_constellatus*, and *Pseudoxanthobacter_liyangensis* were significant indicators for middle-layer beebread. In contrast, the occurrence of *Frischella_perrara*, *Gilliamella_apicola*, and *Paenibacillus_nicotianae* was indicative for substratum-layer beebread.

In order to compare the spatial specificity of microbial distribution in beebread samples under different ecosystems, we estimated indicator species analysis (indicator value *P* < 0.05) of beebread samples for each layer separately (Fig. S5). For the upper beebread, *Aeromonas_hydrophila_subsp_hydrophila* was the significant indicator for forest beebread; *Exiguobacterium_indicum*, *Ruminococcus_flavefaciens*, and *Sphingomonas_humi* were significant indicators for urban-farmland beebread. *Bartonella_apis* and *Megalopta_genalis* were significant indicators for middle-layer beebread collected from forest-urban and urban-farmland colonies, respectively. In the substratum beebread samples, significantly higher relative abundance of *Frischella_perrara* and *Gilliamella_apicola* was found in forest area bee colonies. *Megalopta_genalis* and *Lactobacillus_kunkeei* were significant indicators of beebread from forest-urban bee colonies. *Paenibacillus_sp_CC-1XP5* was significant indicator for substratum beebread from urban-farmland colonies. No significant indicator species were found in the upper-layer beebread of forest-urban area and the middle-layer beebread of forest area.

### Co-occurrence network analysis

**FIG 6:**
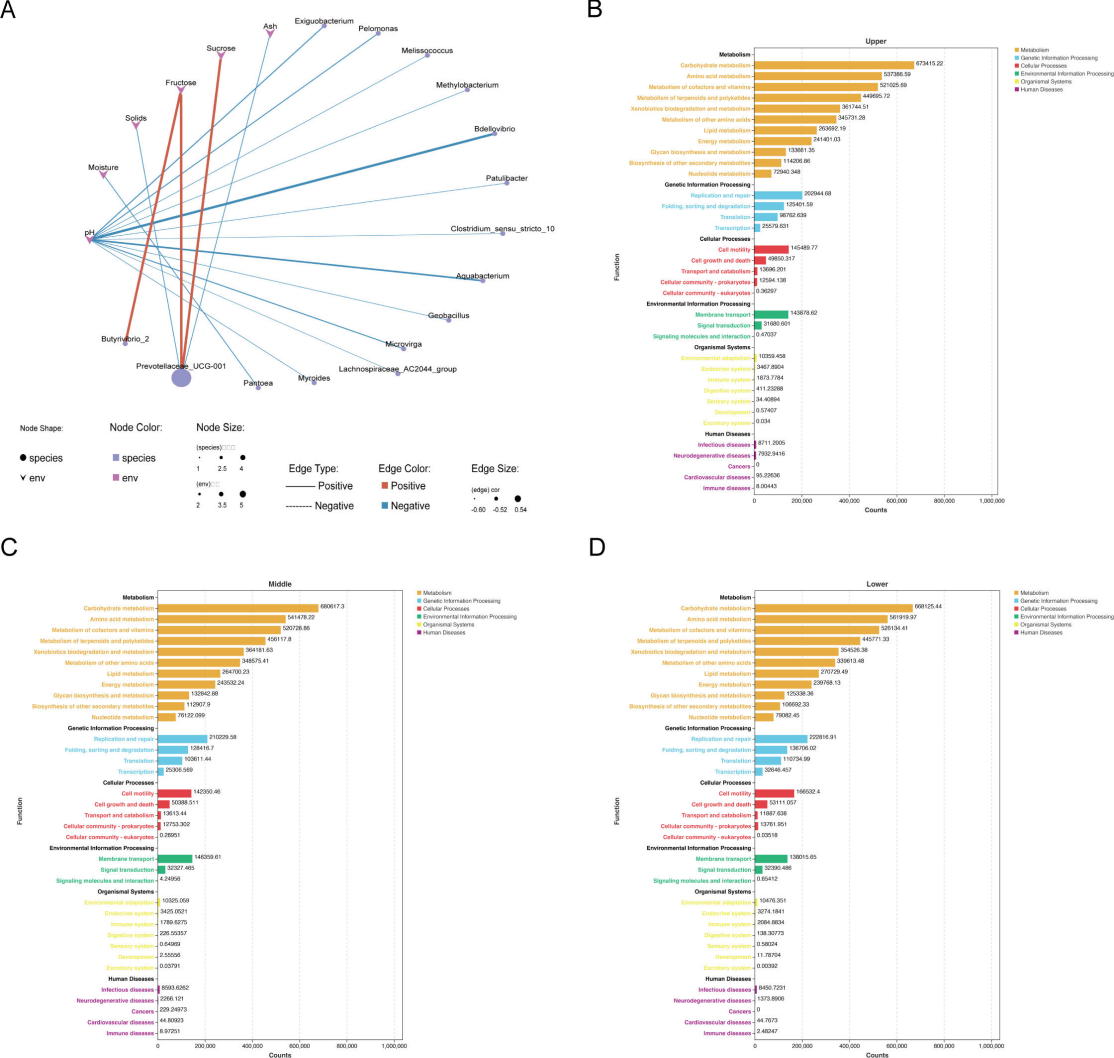
Co-occurrence network analysis and PICRUSt2-based microbial function analysis. (**A**) Pearson correlation analysis of microbial genera in beebread and physicochemical characteristics (*P* < 0.01). (B–D) Microbial functions in different beebread layers.

The correlation between chemical components of beebread and microbial genera is shown in Fig. 6A and Table S10. The relative abundance of *Butyrivibrio_2* was significantly positively correlated with the fructose content of beebread (*P* < 0.01). The relative abundance of *Prevotellaceae_UCG-001* was significantly positively correlated with the contents of fructose and sucrose in beebread (*P* < 0.01) and significantly negatively correlated with the contents of solids and ash. The relative abundance of *Pantoea* was negatively correlated with the moisture content of beebread. The relative abundance of *Bdellovibrio*, *Aquabacterium*, *Exiguobacterium*, *Microvirga*, *Pelomonas*, *Patulibacter*, *Lachnospiraceae_ AC2044_ group*, *Clostridium_ sensu_ stricto_ 10*, *Geobacillus*, *Myroides*, *Metallobacterium*, and *Melissoccus* was significantly negatively correlated with the pH of beebread (*P* < 0.01).

### Microbial function prediction analysis

To evaluate the functional differences of microbial communities in beebread at different depths, the PICRUSt2-based predicted functional analysis was conducted. The Kyoto Encyclopedia of Genes and Genomes database was used to compare the sequencing data and all samples involved into six types of biological metabolic pathways: metabolism, genetic information processing, cellular processes, environmental information processing, organismal systems, and human diseases (Fig. S6B through D). Among them, metabolism was the most dominate function, with abundance of 80.80%, 80.75%, and 79.74%, respectively. Further analysis of the secondary functional level of the prediction gene revealed 35 subfunctions, including carbohydrate metabolism, amino acid metabolism, and metabolism of cofactors and vitamins (Table S11). In all the secondary subfunctional genes, except for low-abundance functional genes of neurodegenerative diseases, digestive system, and development, in which there were significant differences between different groups, the other functional genes have no significant differences (Table S11).

## DISCUSSION

The question of how bees (which have relatively simple but highly specialized intestinal microbiota) obtain the required microbiota from the natural environment (which is inhabited by complex microbial communities) is an important scientific issue in the field of microbial ecology. Beebread is an important source of bee intestinal microbes (5), and the mature beebread in each honeycomb cell is known to exhibit typical spatial distribution patterns. Studies on beebread microbes have mainly focused on the evaluation of overall samples of beebread from honeycombs (5, 16, 17), but there is little knowledge about the vertical distribution patterns of beebread microbes in the honeycomb cells. Here, our objective was to describe, compare, and understand the vertical distribution of these microbes. We collected beebread samples from three ecosystem types for comparative analysis. Our research will help to improve the understanding of the spatial distribution of beebread microbes and provide insights into the diversity and stability of bee microbes.

We also found that the physicochemical characteristics (including nutrient composition) of the upper beebread were significantly different from those of the lower beebread, displaying typical stratification along the vertical axis. Although it has been known for a long time that beebread in honeycombs varies along the vertical axis, this may be the first study to reveal the vertical distribution patterns at the physicochemical and microbial levels. Comparing the physicochemical characteristics of different layers of beebread, we found that there were obvious differences in beebread processing at the upper vs lower layers. The upper beebread contained a higher level of carbohydrate and lower levels of total solids, protein, and ash, which means more honey and less pollen. In contrast, beebread in the middle and lower layers contained more pollen and less honey. The levels of protein, fructose, glucose, and ash in the middle and lower layers were consistent with the findings of other studies (5, 30 – 34). However, we found a higher level of sucrose in the upper layer compared to the lower and middle layers, which is different from previous study (34). We also noticed that the moisture content of our beebread samples was similar to a study by Niu et al. (35) but significantly higher than that of beebread samples collected earlier by Wang et al. (5). This may be related to seasonal differences or more abundant honey and flower sources outside during the current study. In general, honey is more acidic than beebread (5, 23, 36). Therefore, the high acidity of the upper beebread may be due to the higher proportion of honey in upper beebread than lower beebread. A higher proportion of honey is conducive to creating a more tightly sealed environment for anaerobic microbial fermentation of beebread, and a lower pH can also effectively prevent the harmful microbes outside the hive from contaminating the beebread.

Fermentation makes the microbes in the beebread homogenous and stable. As an important pollinator, bees collect pollen that is extremely rich in a large variety of microbial species (21). However, not all of these microbes are used by bees, and only a few kinds of microbes are preserved in beebread. In this study, Proteobacteria, Firmicutes, Actinomyces, and Bacteroides were the four most abundant bacteria in all three layers of all beebread sample groups, which is consistent with a previous study (5). However, there were significant differences in the microbial community structure between the upper and lower layers, indicating that the microbial composition of fermented mature beebread is unstable at the vertical scale. Studies have shown that, across different environments and different seasons, the chemical composition and microbes in beebread of different bee colonies are relatively conserved (5, 16, 17, 23, 30), which is very difficult for small insects like bees. We found that, according to their needs, bees can use simple fermentation processes to customize their “microbial menu,” which is based on the diverse and highly populated external “microbial warehouse,” i.e., flower pollen. Organic acids produced by multiplying microbes may play an important role in beebread fermentation and further shaping the microbial community.

**FIG 7:**
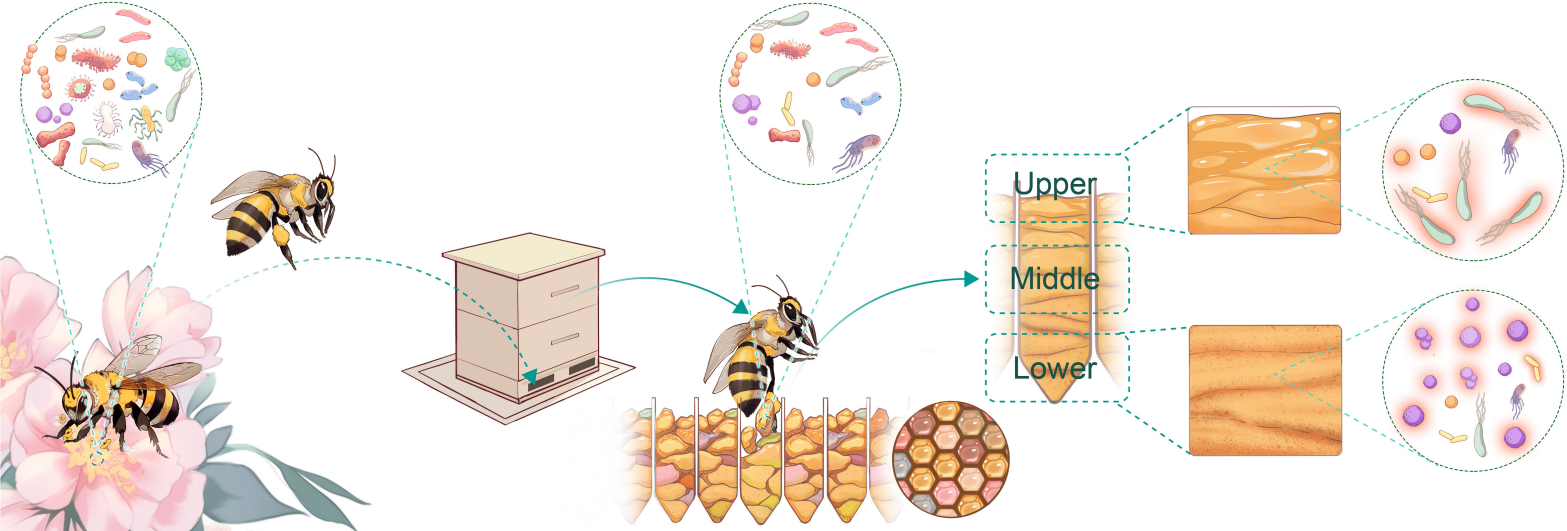
Horizontal transfer of microbes in beebread fermentation process. Flower pollen contains complex and highly abundant microbial communities (21). The number of microbes in bee-collected pollen decreases rapidly as the bees add saliva, enzymes, and nectar (16, 38). The bees unload the pollen into the hive, compact it, and then apply a thin layer of honey to the surface of the pollen, which makes the nutrient content at the surface different from that beneath it. Under the right temperature, humidity, and osmotic pressure conditions in the hive, the microbes in the pollen begin to ferment the pollen and the pH rapidly decreases, so that the mature beebread usually contains only a few species of microbes in very low abundance (5, 16, 17). Not only did the chemical composition and microbial communities of the upper layer of beebread differ significantly from those of the lower layer, but the microbes in the same layer also differed according to ecosystem type.

We believe that our findings complement the current understanding of the microbial community alterations during the beebread fermentation process (Fig. 7). After the pollen-source plants have flowered, the stamens exposed to the air are contaminated by airborne microbes. The contaminated pollen contains a large number of microbes (4 × 10^5^ to 7 × 10^8^) (26). After bees collect the pollen, they add saliva, enzymes, and other substances to the pollen. This raises the pH of the pollen (5.05–5.92) (13, 37). Under the influence of these substances, the number of microbes in the pollen seems to be reduced (4.5 × 10^3^ to 2.5 × 10^5^) (16, 38). The bees transfer the pollen back to the nest and compact it. Under the right temperature, humidity, and osmotic pressure conditions, the microbes in the pollen, especially lactic acid bacteria, begin to multiply rapidly. The organic acids produced by these microbes increase and the pH rapidly decreases (3.92–4.22) (5, 23, 37), inhibiting and killing many acid-sensitive microbes. The microbial community structure of the mature beebread is reshaped (5, 16). Notably, our findings and those of other researchers show that the low pH of beebread can effectively prevent the excessive growth of harmful microbes, thereby stopping spoilage and improving the storage of beebread.

The processing of bee-collected pollen (which involves low pH and moisture, high osmotic pressure, and a tightly sealed environment) may play a crucial role in the selection of beebread microbes. Diet strongly influences the formation of the intestinal microbial structure (39 – 41). Beebread is an important source of protein for bees, and it contains the dominant microbes that are found in the bee intestine (5). This study and previous studies have shown that although beebread has typical microbial structure characteristics, the number of microbes in beebread is very small (hundreds of OTUs). Nevertheless, our results show that the microbial structure (taxa and quantity) in beebread is significantly affected by ecosystem type. Whether this leads to differences in the intestinal microbiota of bees in different ecosystem types requires further study. The relatively conserved microbial structure of beebread is conducive to the stability of the bee intestinal microbiota and the health of bees. Flower pollen and bee-collected pollen have complex compositions and large quantities of microbes, and they can contain many pathogenic microbes that are harmful to bees (17). If bees feed directly from these pollens, the microbes are bound to have a significant impact on the stability of the bee intestinal microbiota, leading to disturbances in the microbiota. Our results showed that the microbial functions in mature beebread did not significantly differ by layer or ecological type, indicating that the fermentation process likely played an important role in the sensitive selection of specific microbes. Many harmful microbes were targeted for removal or suppression. Ultimately, the beebread microbial control function was relatively consistent, which is undoubtedly very important for bees owing to their simple digestive system. In addition, our research shows that although the dominant microbes in beebread were relatively stable, the microbial composition of beebread was still significantly affected by ecological type (e.g., plant sources).

Given that food nutrition affects the structure of the bee gut microbiota (5), the gut microbes of bees feeding on the upper layer of beebread may differ from those feeding on the next layer of beebread. The chemical composition of beebread is affected by many factors (42, 43), such as plant source, honeybee variety, and season. It is difficult to find beebread with the same characteristics even in the same colony (44). Therefore, we adopted a nine-point sampling method to avoid sampling issues as much as possible. It should be noted that although the physicochemical characteristics of the upper beebread were different from those of the lower beebread, generally, the upper beebread only accounts for a small portion of all beebread. When the bee colony needs to rear numerous larvae or new bees, the beebread is rapidly consumed, especially during periods of honey and flower scarcity, making it challenging to gather a complete sample of mature beebread, including the upper layer. In any case, our results change the understanding of the consistency of the nutrient composition of beebread. Our results indicate that bees filter out the many microbes in their pollen, conserving the overall microbial diversity among beebread samples. This may be the secret of how bees, which have a simple digestive tract and simple microbial composition, survive and thrive in different ecosystems. Of course, the mechanisms that determine the intestinal microbes of bees may be multifaceted, and this warrants further in-depth study.

Moreover, this study focuses on the vertical distribution of beebread microbial communities within specific ecosystem bee colonies. Throughout the experimental process, efforts were made to minimize external influences; however, there are still certain limitations to be acknowledged in this research. Firstly, previous studies have indicated a high degree of similarity in the community structure of beebread microbial populations within different bee colonies in the same habitat (5, 16). In this study, three samples were utilized for each treatment in every ecosystem, following strict sampling protocols. However, increasing the sample size undoubtedly improves the validity of the results. Secondly, the microbial composition of beebread may be influenced by various factors such as environmental conditions, seasons, and fermentation time. Considering the unique nature of our research and the regional limitations of the investigated environments, conducting similar studies in different regions and diverse environments in the future will contribute to a better understanding of how the environment influences the physicochemical properties and distribution patterns of beebread microbial communities. Despite these limitations, we believe our results provide valuable insights into the beebread microbial communities of these ecosystems.

This study systematically investigated the vertical distribution patterns in honeycomb cells of both the physicochemical characteristics of beebread and the microbial community. The results showed that the physicochemical characteristics (pH, total solids, crude protein, fructose, sucrose, and ash) of the upper layer of beebread were significantly different from those of the lower layer. The 16S rRNA gene sequencing results showed that the microbial community structure of different beebread layers varied not only at the vertical scale but also by ecosystem type. Our research suggests that complex microbial changes during the fermentation process of beebread (which makes beebread more easily digested by bees than pollen) lead to the remodeling and relative stability of the microbial communities, which are very important for the health of bees.

## MATERIALS AND METHODS

### Beebread sampling

Samples of beebread were collected from three apiaries in Tai’an, Shandong province, P.R. China (Fig. S6). Details regarding the three sample collection sites are shown in Table S12. Based on bees’ effective pollen collection radius of 2.5 km, the ecosystem types of the three apiaries were classified as forest, forest-urban, and urban-farmland.

**FIG 8:**
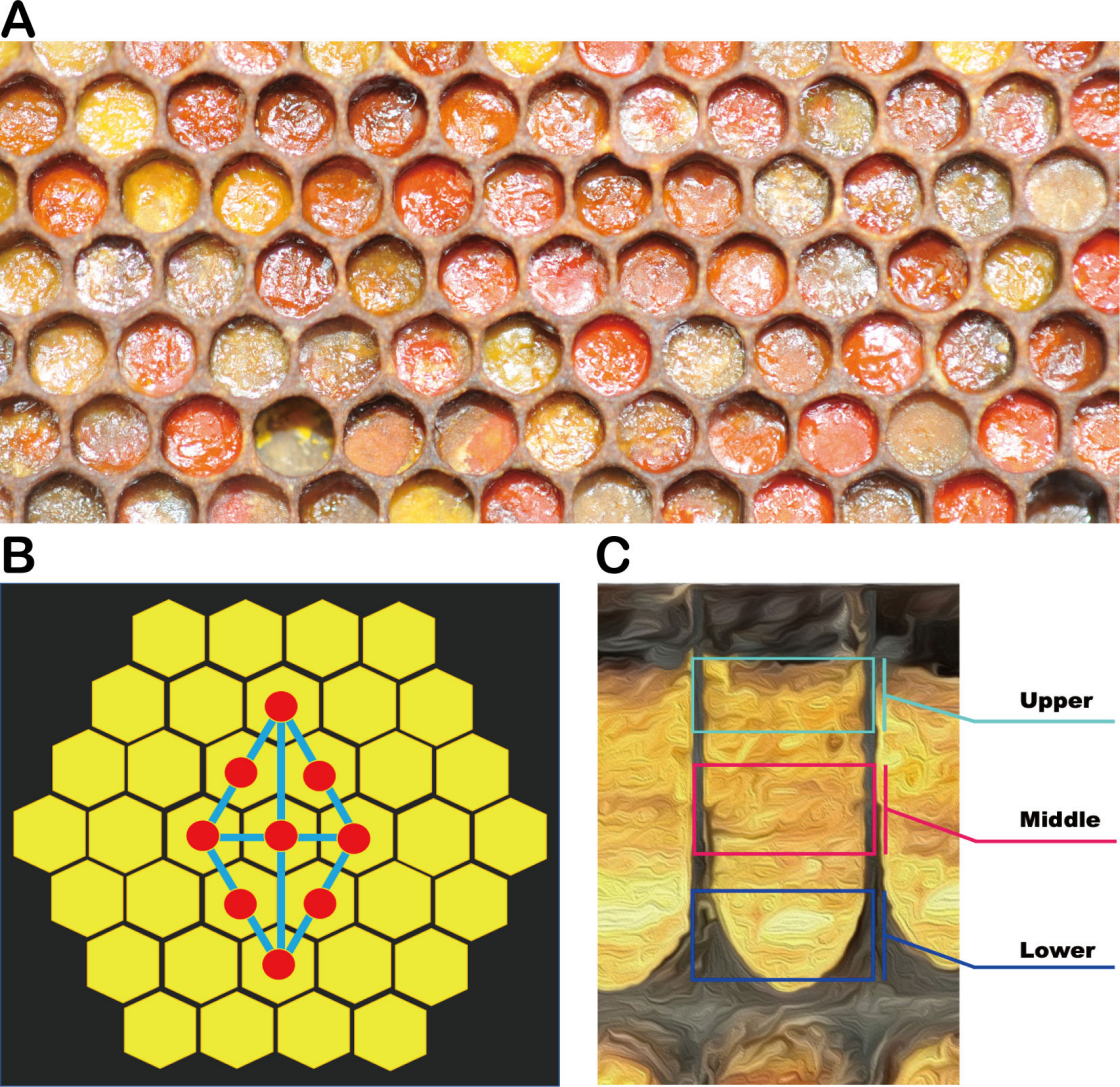
Sampling strategy. (**A**) Honeycomb with stored beebread; once the hive is filled with pollen, the bees apply a layer of honey, so the upper beebread has a shinier surface. (**B**) Nine-point sampling method used for each colony. (**C**) Section diagram of beebread in honeycombs. The beebread was divided into upper, middle, and lower layers, respectively.

In September 2020, three *Apis mellifera* L. colonies were randomly selected for each apiary, and an empty comb was introduced into each colony. The beebread sampling was carried out in October 2020 according to a nine-point sampling method (Fig. 8B). The cells were cut longitudinally with a blade, and the beebread samples were divided into three subsamples: upper, middle, and lower (Fig. 8C). The beebread subsamples were collected with sterilized disposable plastic sheets. In total, 27 beebread samples (three subsamples × three colonies × three apiaries) were obtained. The beebread samples were immediately stored in dry ice, transported to the laboratory, and stored at −80°C for physicochemical analysis and DNA extraction.

### Physicochemical characteristics analysis

To determine the physicochemical characteristics of beebread, 0.5 g (to the nearest 0.1 mg) of beebread was used for each test. The standard methods in the literature were used to evaluate the beebread pH, moisture, crude protein, fructose, glucose, sucrose (5), total solids (30), and ash (45).

### DNA extraction

Prior to DNA extraction, about 0.5-g beebread was homogenized for 1 min at 12.0 rpm with 10-mL 1× phosphate-buffered saline at 0°C. The genomic DNA was then extracted with Animal Genomic DNA Rapid Extraction Kit (Beyotime) following the manufacturer’s instructions. The DNA extracts were stored at −80°C until they were sent for sequencing.

### 16S rRNA gene sequencing

The DNA extracts were sent on dry ice to GENEDENOVO Biological Technology Co. Ltd. (Guangzhou, China) for sequencing. The bacterial 16S rRNA genes were sequenced using the 799F (46) and 1193R (46) primer pairs on an Illumina platform. FASTP (version 0.18.0) (47) was used to perform quality filtering of the original data to remove bases with an adapter and tail mass value of <20. Thereafter, FLASH (version 1.2.11) (48) was used to splice double-ended reads, and UCHIME (version 9.2.64) (49) was used to remove chimeras, ultimately producing high-quality sequences for subsequent analysis. A cluster with a similarity of >97% was defined as an OTU. Species annotation was then performed using the SILVA bacterial 16S rRNA database (release 132, http://www.arb-silva.de) to obtain OTU taxonomic annotation results for each sample. The raw reads were deposited in the National Center for Biotechnology Information Sequence Read Archive database (BioProject ID: PRJNA961347).

### Statistical analysis

Bioinformatic analysis was performed using Omicsmart (http://www.omicsmart.com), a dynamic real-time interactive online platform for data analysis. Alpha-diversity analysis of the bacterial communities in beebread involved calculating ACE, Chao1, Shannon index, Simpson index, Good’s coverage, and Pielou’s evenness index in QIIME (version 1.9.1) (50) and PD-tree index in the picante R package (version 1.8.2) (51). Beta-diversity analysis was carried out using PCoA and Adonis (PERMANOVA). Indicator species analyses were also conducted. The relative abundances of microbes were compared among groups using Tukey’s honestly significant difference (HSD) test. Tukey’s HSD test and Adonis (PERMANOVA) test were conducted using the Vegan R package (version 2.5.3) (52). The correlations between the relative abundances of beebread microbes and the physicochemical characteristics of beebread (pH, moisture, crude protein, fructose, glucose, sucrose, total solids, and ash) were determined using PICRUSt2 (version 2.1.4) (53). The physicochemical characteristics of beebread were compared among groups using the LSD test or Tukey’s HSD test in SPSS (version 18.0). The data were expressed as mean ± standard deviation. *P* value of < 0.05 was considered statistically significant.

## SUPPLEMENTAL MATERIAL

Supplemental file 1 spectrum.01818-23-s0001.docx Fig. S1 to S6 and Tables S1 to S9, S11, and S12.

Supplemental file 2 spectrum.01818-23-s0002.xlsx Table S10.

ASM does not own the copyrights to Supplemental Material that may be linked to, or accessed through, an article. The authors have granted ASM a non-exclusive, world-wide license to publish the Supplemental Material files. Please contact the corresponding author directly for reuse.
