# Supplementary material for: Bee wisdom: exploring bee control strategies for food microflora by comparing the physicochemical characteristics and microbial composition of beebread
Source: Microbiol Spectr. 2023 Oct 6;11(6):e01818-23. doi: 10.1128/spectrum.01818-23 (PMC10871783; doi:10.1128/spectrum.01818-23)
Supplement: Supplemental file 1 — Fig. S1 to S6 and Tables S1 to S9, S11, and S12. [file spectrum.01818-23-s0001.docx]

**Supplementary information**

**
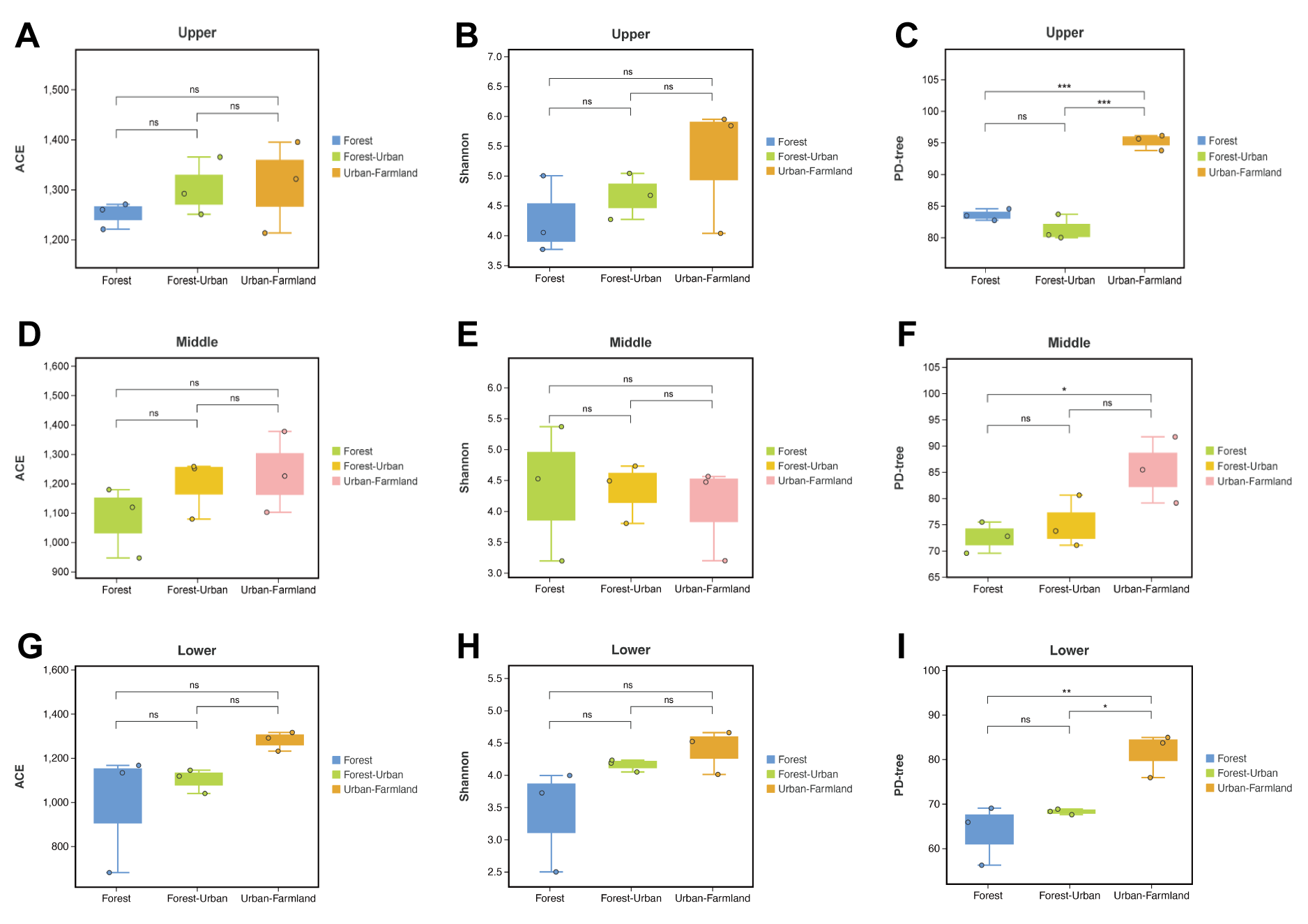
**

**Supplementary Figure S1. Comparison of alpha-diversity indexes of the microbiota in the (A–C) upper, (D–F) middle, and (G–I) lower beebread layers among the three ecosystem types.** Values are mean ± SD. *P<0.05, **P<0.01 and ***P<0.001 based on Tukey’s HSD test.

**
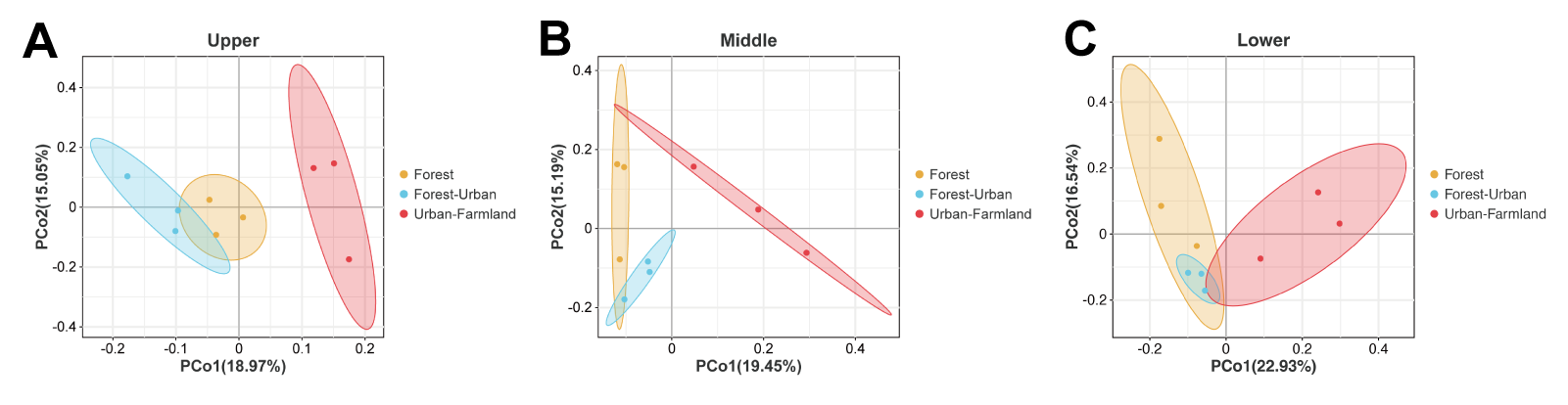
**

**Supplementary Figure S2. Principal coordinate analysis (PCoA) of beebread microbial communities of three ecosystem types and three layers based on unweighted UniFrac dissimilarity matrix (n=3).**

**
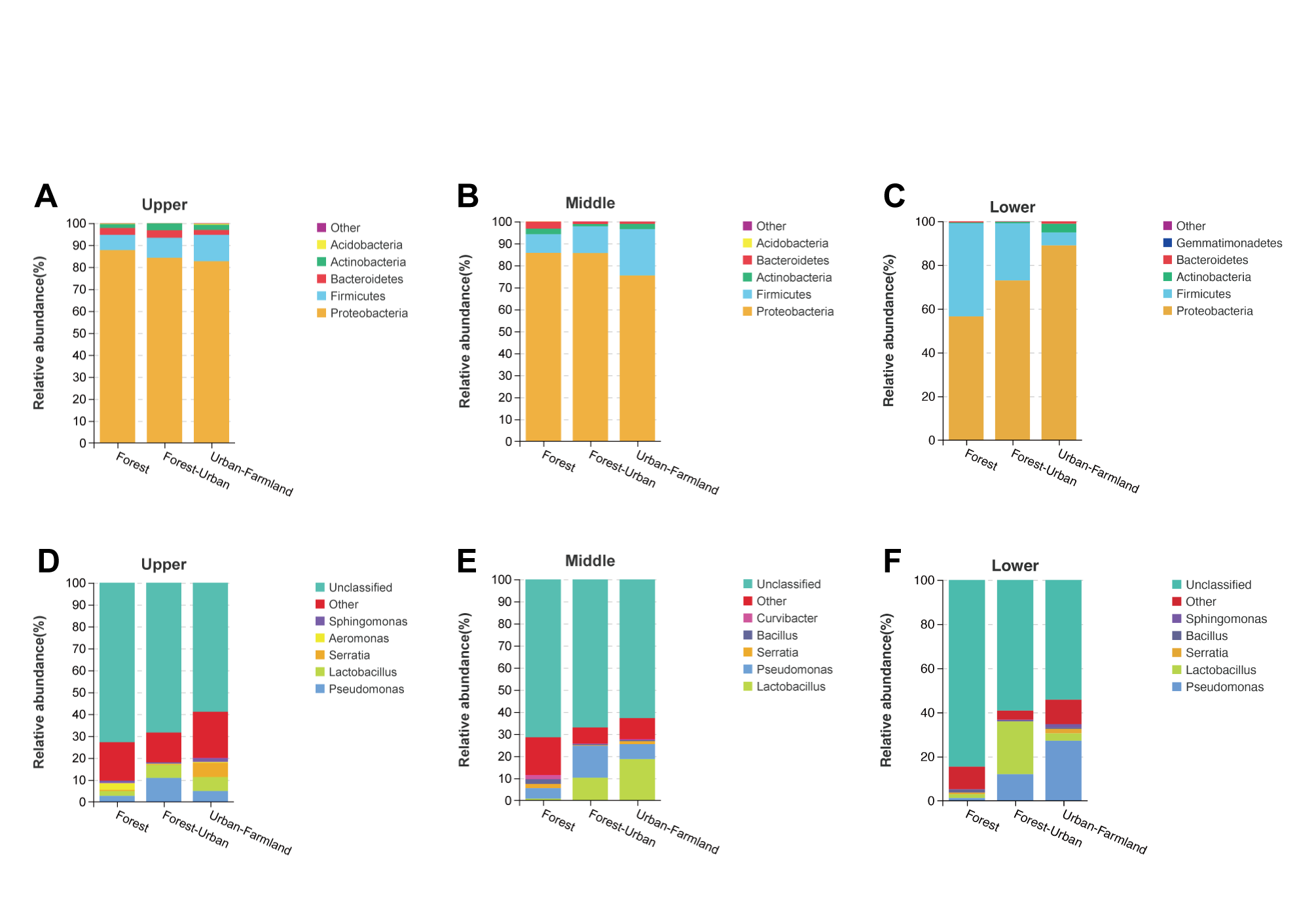
**

**Supplementary Figure S3. Comparison of major bacterial phyla and genera in each beebread layer among the three ecosystem types.** Relative abundances of the five most abundant (A–C) phyla and (D–F) genera.

**
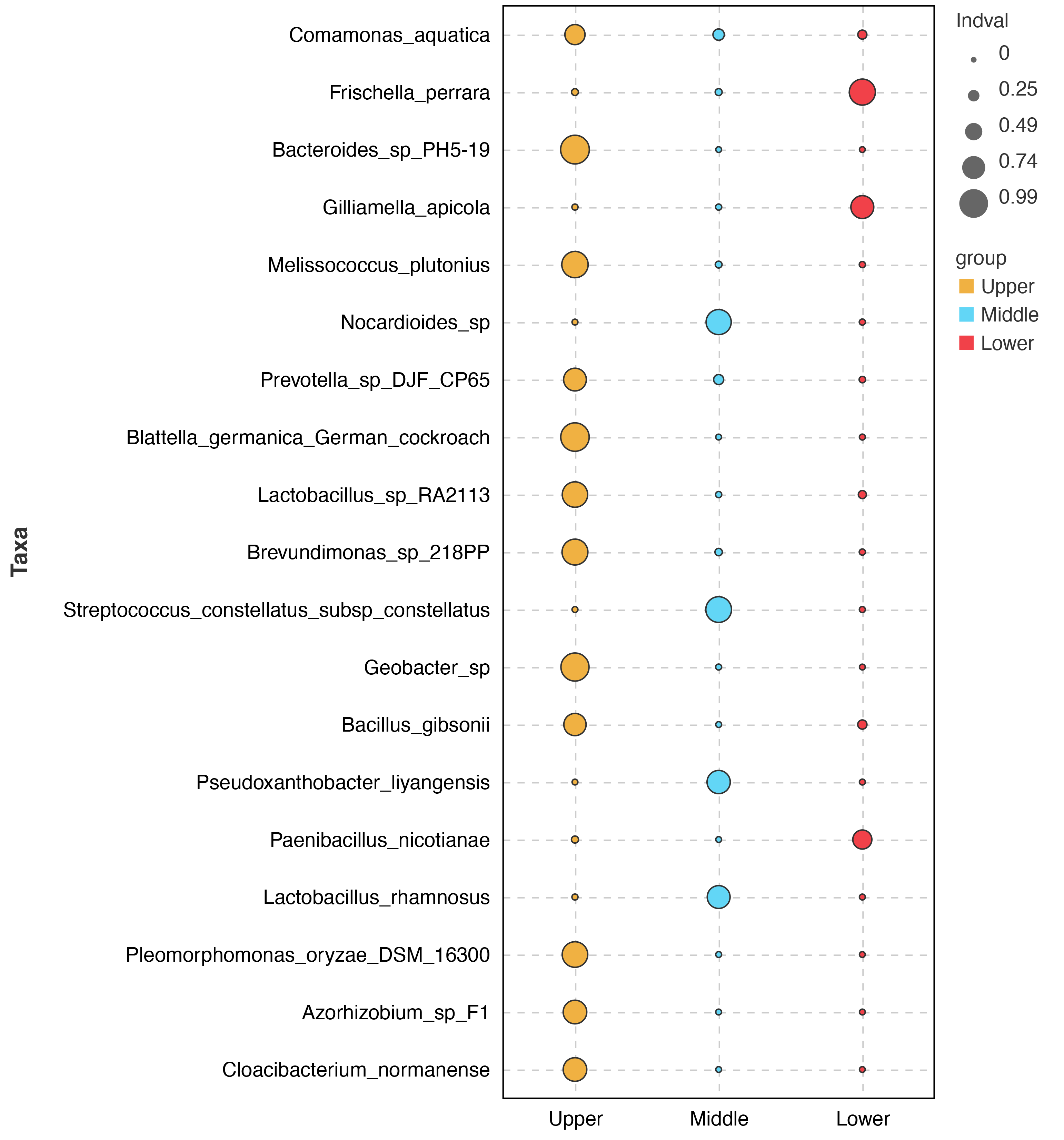
**

**Supplementary Figure S4. 19 most important species for prediction of beebread layer (upper, middle, and lower) based on species abundance and frequency.**

**
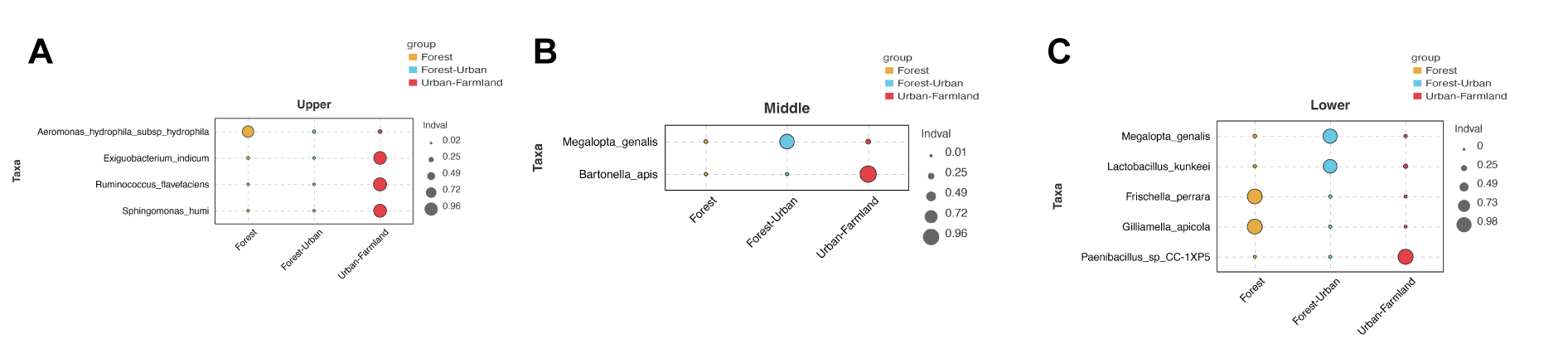
**

**Supplementary Figure S5. Most important species in each beebread layer for the prediction of ecosystem type based on species abundance and frequency.**


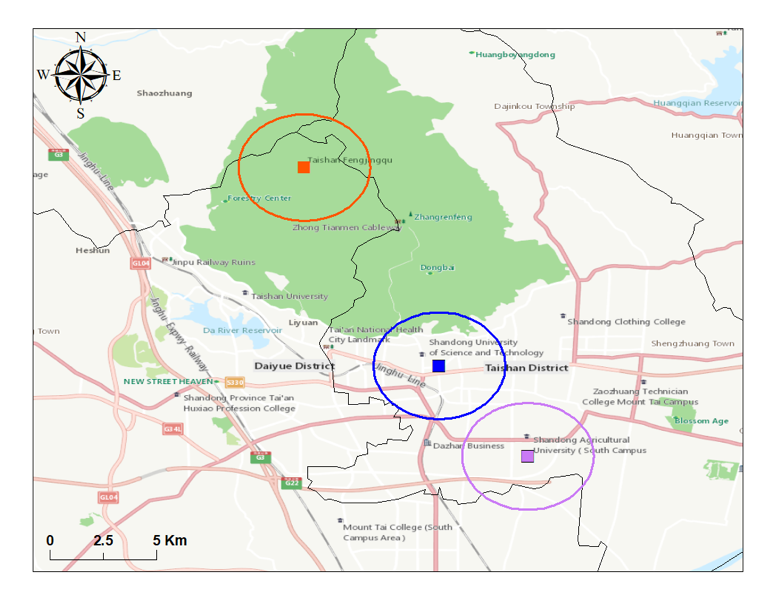


**Supplementary Figure S6 Location of sampling sites.**

**Supplementary Table S1. Microorganisms in flower pollen, bee collected pollen and beebread.** During the process of pollen being collected by bees from flowers and converted into beebread, the number of microorganisms carried in flower pollen decreases dramatically.

| **Item** | **Bee species** | **Bacteria numbers  (CFUs per gram of dry pollen weight)** | **OTUs** | **Dominant bacterial phyla** | **Origin** | **Reference** |
| --- | --- | --- | --- | --- | --- | --- |
| Flower Pollen |  | 4.1 ± 3.1 × 10^5^-7.5 ± 7.4 × 10^8^ | 11668 - 38188 | Proteobacteria Actinobacteria Firmicutes Bacteroidetes | Germany | Ambika et al., 2016 |
|  |  | - | 1032-2013 | Proteobacteria Firmicutes Actinobacteria Acidobacteria Deinococcus-Thermus | Germany |  |
| Bee collected pollen | A. mellifera | 5.0 × 10^1^-2.5  × 10^5^ | 663 |  | China | Biao et al., 2020 |
|  | A. mellifera | 1.1 × 10^4^-2.4  × 10^4^ | 189-325 | Proteobacteria Firmicutes Actinobacteria Bacteroidetes  Deinococcus-Thermus Spirochaetae Fusobacteria | China | Disayathanoowat et al., 2020 |
|  | A. cerana | 4.5 × 10^3^-8.2  × 10^3^ | 153-347 |  |  |  |
| Beebread | A. mellifera | 5.4 × 10^3^-8.9  × 10^3^ | 167-240 | Proteobacteria Firmicutes Actinobacteria Bacteroidetes  Deinococcus-Thermus Spirochaetae Fusobacteria | China |  |
|  | A. cerana | 6.5 × 10^2^-1.4  × 10^3^ | 115-201 |  |  |  |
|  | A. mellifera |  | 522-617 | Proteobacteria  Firmicutes Bacteroidetes  Actinobacteria  Planctomycetes | China | Wang et al., 2021 |
|  | A. cerana |  | 542-489 |  |  |  |

**Supplementary Table S2.** **Physicochemical characteristics of beebread samples.** Values are mean ± SD (n=9). *P<0.05, **P<0.01 and ***P<0.001 based on LSD test.

| **Parameters** | **Contents (%, fresh weight)** | | | **ANOVA** | | **p value** | | |
| --- | --- | --- | --- | --- | --- | --- | --- | --- |
|  | **Upper** | **Middle** | **Lower** | **F** | **P** | **Upper vs Middle** | **Upper vs Lower** | **Middle vs Lower** |
| pH | 4.02±0.11 | 4.24±0.11 | 4.22±0.13 | 9.707 | 0.001 | 0.001 | 0.001 | 0.693 |
| Moisture | 20.62±2.30 | 22.21±2.73 | 21.86±3.11 | 0.847 | 0.441 | 0.228 | 0.343 | 0.789 |
| Crude protein | 22.13±1.03 | 23.94±1.47 | 24.05±1.70 | 5.125 | 0.014 | 0.013 | 0.009 | 0.868 |
| Fructose | 20.95±1.52 | 18.80±0.95 | 19.64±0.52 | 9.07 | 0.001 | 0 | 0.016 | 0.113 |
| Glucose | 10.52±0.64 | 10.00±0.63 | 10.38±0.36 | 2.067 | 0.148 | 0.062 | 0.62 | 0.159 |
| Sucrose | 5.33±0.18 | 4.25±0.19 | 4.49±0.34 | 47.029 | 0 | 0 | 0 | 0.053 |
| Ash | 2.60±0.36 | 3.03±0.33 | 3.02±0.33 | 4.696 | 0.019 | 0.013 | 0.014 | 0.978 |
| Total solids | 29.02±0.91 | 37.93±2.08 | 38.27±2.29 | 71.25 | 0 | 0 | 0 | 0.696 |

**Supplementary Table S3.** **Sequencing output results.**

| **Sample Name** | **Ecological type** | **Raw PE** | **Clean PE** | **Raw Tags** | **Clean Tags** | **Effective Tags** | **Effective Ratio (%)** | **Unique Tags** | **Taxon Tags** | **Unclassified Tags** | **Singleton Tags** | **OTUs** |
| --- | --- | --- | --- | --- | --- | --- | --- | --- | --- | --- | --- | --- |
| Upper-1 | Urban-Farmland | 126754 | 126685 | 124428 | 122760 | 108484 | 85.59 | 26350 | 91703 | 0 | 16781 | 904 |
| Upper-2 | Urban-Farmland | 137610 | 137518 | 135352 | 133561 | 122523 | 89.04 | 28317 | 105262 | 0 | 17261 | 937 |
| Upper-3 | Urban-Farmland | 120743 | 120654 | 118351 | 116357 | 97607 | 80.84 | 24428 | 80239 | 0 | 17368 | 931 |
| Upper-4 | Forest | 128953 | 128854 | 126855 | 125327 | 110846 | 85.96 | 22004 | 91753 | 0 | 19093 | 828 |
| Upper-5 | Forest | 126324 | 126230 | 124273 | 122486 | 110841 | 87.74 | 22624 | 98841 | 0 | 12000 | 840 |
| Upper-6 | Forest | 123816 | 123737 | 121745 | 120062 | 107282 | 86.65 | 23023 | 93266 | 0 | 14016 | 809 |
| Upper-7 | Forest-Urban | 135827 | 135753 | 133722 | 131799 | 119693 | 88.12 | 24423 | 107897 | 0 | 11796 | 842 |
| Upper-8 | Forest-Urban | 126464 | 126378 | 124755 | 123095 | 114373 | 90.44 | 23960 | 103625 | 0 | 10748 | 803 |
| Upper-9 | Forest-Urban | 121533 | 121437 | 119162 | 117479 | 108551 | 89.32 | 24064 | 97581 | 0 | 10970 | 781 |
| Middle-1 | Urban-Farmland | 135250 | 135154 | 132864 | 130914 | 118598 | 87.69 | 23860 | 107438 | 0 | 11160 | 850 |
| Middle-2 | Urban-Farmland | 130874 | 130780 | 128514 | 126747 | 111403 | 85.12 | 25902 | 88608 | 0 | 22795 | 909 |
| Middle-3 | Urban-Farmland | 130607 | 130532 | 128669 | 126819 | 111253 | 85.18 | 22357 | 77307 | 0 | 33946 | 812 |
| Middle-4 | Forest | 128407 | 128315 | 126083 | 124020 | 110193 | 85.82 | 23184 | 91842 | 0 | 18351 | 783 |
| Middle-5 | Forest | 132034 | 131936 | 130181 | 128584 | 114291 | 86.56 | 24191 | 100362 | 0 | 13929 | 671 |
| Middle-6 | Forest | 135080 | 135002 | 132953 | 131179 | 116839 | 86.5 | 24621 | 102435 | 0 | 14404 | 733 |
| Middle-7 | Forest-Urban | 122344 | 122255 | 120404 | 118783 | 102337 | 83.65 | 22466 | 90522 | 0 | 11815 | 803 |
| Middle-8 | Forest-Urban | 126086 | 126015 | 124095 | 122259 | 106405 | 84.39 | 24249 | 93760 | 0 | 12645 | 734 |
| Middle-9 | Forest-Urban | 136335 | 136261 | 134487 | 132761 | 121723 | 89.28 | 25080 | 110571 | 0 | 11152 | 799 |
| Lower-1 | Urban-Farmland | 135326 | 135221 | 133262 | 131439 | 100482 | 74.25 | 30172 | 77275 | 0 | 23207 | 822 |
| Lower-2 | Urban-Farmland | 133834 | 133742 | 131784 | 129796 | 117466 | 87.77 | 23448 | 103092 | 0 | 14374 | 792 |
| Lower-3 | Urban-Farmland | 136629 | 136536 | 133698 | 131806 | 116426 | 85.21 | 26309 | 94546 | 0 | 21880 | 862 |
| Lower-4 | Forest | 121561 | 121495 | 119782 | 118135 | 109584 | 90.15 | 20250 | 81973 | 0 | 27611 | 680 |
| Lower-5 | Forest | 133864 | 133773 | 131538 | 129445 | 116879 | 87.31 | 24029 | 100796 | 0 | 16083 | 693 |
| Lower-6 | Forest | 125663 | 125588 | 123920 | 122714 | 113721 | 90.5 | 19135 | 105921 | 0 | 7800 | 512 |
| Lower-7 | Forest-Urban | 123840 | 123774 | 121982 | 120368 | 105572 | 85.25 | 22093 | 92285 | 0 | 13287 | 672 |
| Lower-8 | Forest-Urban | 122397 | 122317 | 120405 | 118707 | 107103 | 87.5 | 21490 | 97087 | 0 | 10016 | 715 |
| Lower-9 | Forest-Urban | 123350 | 123275 | 121161 | 119404 | 108928 | 88.31 | 22189 | 98535 | 0 | 10393 | 692 |
| Total |  | 3481505 | 3479217 | 3424425 | 3376806 | 3009403 |  | 644218 | 2584522 | 0 | 424881 | 21209 |

**Supplementary Table S4. Comparison of alpha-diversity indexes of beebread samples based on Tukey’s HSD test (n=3).**

| **Index** | **p value** | | |
| --- | --- | --- | --- |
|  | **Middle vs Upper** | **Lower vs Upper** | **Lower vs Middle** |
| Good's coverage | 0.428 | 0.399 | 0.998 |
| ACE | 0.181 | 0.044 | 0.748 |
| Chao1 | 0.227 | 0.135 | 0.952 |
| Shannon | 0.349 | 0.087 | 0.698 |
| Simpson | 0.828 | 0.808 | 0.999 |
| PD-tree | 0.052 | 0.001 | 0.187 |

**Supplementary Table S5. Comparison of alpha-diversity indexes of the microbiota of three ecosystem types and three beebread layers using Tukey’s HSD test (n=3).**

| **Layers** | **Index** | **p value** | | |
| --- | --- | --- | --- | --- |
|  |  | **p(Forest-Urban_vs_Forest)** | **p(Urban-Farmland_vs_Forest)** | **p(Urban-Farmland_vs_Forest-Urban)** |
| Upper | ACE | 0.608 | 0.531 | 0.989 |
|  | Chao1 | 0.717 | 0.274 | 0.649 |
|  | Shonnon | 0.811 | 0.309 | 0.608 |
|  | Simpson | 0.352 | 0.462 | 0.968 |
|  | PD-tree | 0.235 | 0.000 | 0.000 |
| Middle | ACE | 0.517 | 0.334 | 0.919 |
|  | Chao1 | 0.381 | 0.217 | 0.887 |
|  | Shonnon | 0.999 | 0.906 | 0.919 |
|  | Simpson | 0.759 | 0.980 | 0.652 |
|  | PD-tree | 0.808 | 0.043 | 0.095 |
| Lower | ACE | 0.710 | 0.157 | 0.421 |
|  | Chao1 | 0.749 | 0.096 | 0.247 |
|  | Shonnon | 0.240 | 0.114 | 0.832 |
|  | Simpson | 0.048 | 0.091 | 0.864 |
|  | PD-tree | 0.518 | 0.009 | 0.034 |

**Supplementary Table S6. Comparison of the relative abundances of the five most abundant bacterial phyla in beebread samples of different layers.** Values are mean ± SD (n=9). Different letters in the same column (a and b) indicate a significant difference at P<0.05 based on Tukey’s HSD test.

|  | **Proteobacteria** | **Firmicutes** | **Actinobacteria** | **Bacteroidetes** | **Acidobacteria** |
| --- | --- | --- | --- | --- | --- |
| Upper | 84.87±6.45a | 9.33±5.25a | 2.45±1.72a | 2.93±2.24a | 0.28±0.37a |
| Middle | 82.27±17.23a | 13.87±15.17a | 2.00±1.81a | 1.76±2.64ab | 0.04±0.05b |
| Lower | 72.83±24.72a | 24.89±25.88a | 1.65±1.96a | 0.60±0.67b | 0.00±0.01b |
| F | 1.141 | 1.865 | 0.438 | 2.973 | 4.471 |
| p | 0.336 | 0.177 | 0.65 | 0.07 | 0.022 |

**Supplementary Table S7. Comparison of the relative abundances of the five most abundant bacterial genera in beebread samples of different layers.** Values are mean ± SD (n=9). Different letters in the same column indicate a significant difference at P<0.05 based on Tukey’s HSD test.

|  | ***Pseudomonas*** | ***Lactobacillus*** | ***Serratia*** | ***Bacillus*** | ***Sphingomonas*** |
| --- | --- | --- | --- | --- | --- |
| Upper | 6.25±4.11a | 9.23±14.34a | 2.26±3.94a | 1.12±0.78a | 1.18±0.70a |
| Middle | 9.74±7.53a | 4.73±4.87a | 1.57±1.87a | 0.99±1.46a | 0.66±0.30a |
| Lower | 20.43±25.45a | 11.66±13a | 0.49±0.59a | 0.75±0.86a | 0.63±0.79a |
| F | 2.195 | 0.823 | 1.094 | 0.282 | 2.276 |
| p | 0.132 | 0.451 | 0.35 | 0.756 | 0.124 |

**Supplementary Table S8. Comparison of the relative abundances of the five most abundant bacterial phyla in beebread layers from different ecosystem types.** Values are mean ± SD (n=9). Different letters in the same column (a and b) indicate a significant difference at P<0.05 based on Tukey’s HSD test.

| **Layer** | **Group** | **Forest** | **Forest-Urban** | **Urban-Farmland** | **F** | **p** |
| --- | --- | --- | --- | --- | --- | --- |
| Upper | Proteobacteria | 87.72±3.55 | 84.25±6.52 | 82.63±9.53 | 0.416 | 0.678 |
|  | Firmicutes | 6.93±2.70 | 9.02±4.81 | 12.04±7.75 | 0.657 | 0.552 |
|  | Bacteroidetes | 3.11±1.49 | 3.47±3.49 | 2.23±2.10 | 0.193 | 0.829 |
|  | Actinobacteria | 1.83±0.65 | 3.16±2.72 | 2.36±1.63 | 0.381 | 0.698 |
|  | Acidobacteria | 0.29±0.26 | 0.01±0.01 | 0.55±0.51 | 1.971 | 0.22 |
| Middle | Proteobacteria | 85.73±14.06 | 85.66±5.69 | 75.42±29.19 | 0.293 | 0.756 |
|  | Firmicutes | 8.47±7.59 | 12.08±4.50 | 21.06±26.76 | 0.476 | 0.643 |
|  | Actinobacteria | 2.50±2.00 | 1.06±0.89 | 2.44±2.51 | 0.54 | 0.608 |
|  | Bacteroidetes | 3.27±4.59 | 1.11±1.03 | 0.90±0.75 | 0.682 | 0.541 |
|  | Acidobacteria | 0.03±0.04 | 0.05±0.07 | 0.05±0.03 | 0.108 | 0.899 |
| Lower | Proteobacteria | 56.52±37.71 | 72.98±14.46 | 88.99±4.73 | 1.435 | 0.309 |
|  | Firmicutes | 42.63±38.08 | 26.17±14.24 | 5.87±2.80 | 1.837 | 0.239 |
|  | Actinobacteria | 0.3±0.25c | 0.61±0.41b | 4.02±1.51a | 15.335 | 0.004 |
|  | Bacteroidetes | 0.54±0.3 | 0.17±0.15 | 1.09±1.01 | 1.668 | 0.266 |
|  | Gemmatimonadetes | 0±0 | 0.06±0.1 | 0±0 | 1.025 | 0.414 |

**Supplementary Table S9. Comparison of the relative abundances of the five most abundant bacterial genera in beebread samples from different ecosystem types.** Values are mean ± SD (n=9). Different letters in the same column indicate a significant difference at P<0.05 based on Tukey’s HSD test.

| **Layer** | **Group** | **Forest** | **Forest-Urban** | **Urban-Farmland** | **F** | **p** |
| --- | --- | --- | --- | --- | --- | --- |
| Upper | *Pseudomonas* | 2.65±0.60c | 10.89±3.98a | 4.92±2.23b | 7.701 | 0.022 |
|  | *Lactobacillus* | 2.29±1.26 | 5.91±4.93 | 6.34±4.47 | 0.975 | 0.43 |
|  | *Serratia* | 0.47±0.28 | 0.30±0.18 | 6.48±5.61 | 3.534 | 0.097 |
|  | *Aeromonas* | 3.07±1.64a | 0.22±0.10b | 0.39±0.25b | 8.334 | 0.019 |
|  | *Sphingomonas* | 1.18±0.65 | 0.66±0.54 | 1.79±0.70 | 2.412 | 0.17 |
| Middle | *Pseudomonas* | 4.75±4.23 | 14.44±9.68 | 6.73±2.90 | 1.966 | 0.22 |
|  | *Lactobacillus* | 0.78±0.06 | 10.27±3.83 | 18.69±26.08 | 1.04 | 0.409 |
|  | *Serratia* | 1.87±2.72 | 0.30±0.28 | 1.36±0.57 | 0.736 | 0.518 |
|  | *Bacillus* | 2.15±2.31 | 0.48±0.36 | 0.68±0.45 | 1.315 | 0.336 |
|  | *Curvibacter* | 1.92±2.02 | 0.26±0.12 | 0.22±0.16 | 2.06 | 0.208 |
| Lower | *Pseudomonas* | 1.25±0.61c | 12.04±6.87b | 27.20±9.26a | 11.472 | 0.009 |
|  | *Lactobacillus* | 1.75±0.55b | 23.80±14.50a | 3.33±1.38b | 6.406 | 0.032 |
|  | *Serratia* | 0.74±0.82 | 0.10±0.08 | 1.99±2.09 | 1.647 | 0.269 |
|  | *Bacillus* | 1.25±1.32 | 0.39±0.17 | 0.64±0.74 | 0.773 | 0.503 |
|  | *Sphingomonas* | 0.23±0.15b | 0.35±0.20b | 1.55±0.79a | 7.06 | 0.027 |

**Supplementary Table S11. Comparison of PICRUSt2-based microbial function prediction (levels 1 and 2) of different beebread layers.**

| **Level_1** | **Level_2** | **Layers** | | | **F** | **P** | **p value** | | |
| --- | --- | --- | --- | --- | --- | --- | --- | --- | --- |
|  |  | **Upper** | **Middle** | **Lower** |  |  | **Lower Vs Middle** | **Upper vs Middle** | **Upper vs Lower** |
| Metabolism | Carbohydrate metabolism | 673415.22±105345.81 | 680617.30±105819.62 | 668125.44±92824.02 | 0.034 | 0.966 | 0.963 | 0.988 | 0.993 |
| Metabolism | Amino acid metabolism | 537386.59±88695.50 | 541478.22±94892.79 | 561919.97±96421.69 | 0.178 | 0.838 | 0.889 | 0.995 | 0.844 |
| Metabolism | Metabolism of cofactors and vitamins | 521025.69±79647.76 | 520728.86±77026.66 | 526134.41±73822.86 | 0.014 | 0.986 | 0.988 | 1.000 | 0.989 |
| Metabolism | Metabolism of terpenoids and polyketides | 449695.72±86612.50 | 456117.80±90889.46 | 445771.33±124871.43 | 0.023 | 0.977 | 0.975 | 0.990 | 0.996 |
| Metabolism | Xenobiotics biodegradation and metabolism | 361744.51±60319.87 | 364181.63±71910.32 | 354526.38±106263.25 | 0.034 | 0.967 | 0.966 | 0.998 | 0.981 |
| Metabolism | Metabolism of other amino acids | 345731.28±53793.34 | 348575.41±57181.89 | 339613.48±51654.89 | 0.064 | 0.938 | 0.935 | 0.993 | 0.969 |
| Metabolism | Lipid metabolism | 263692.19±45611.62 | 264700.23±57680.23 | 270729.49±72533.27 | 0.037 | 0.964 | 0.975 | 0.999 | 0.966 |
| Metabolism | Energy metabolism | 241401.03±36254.73 | 243532.24±35638.14 | 239768.13±30835.52 | 0.027 | 0.973 | 0.971 | 0.990 | 0.994 |
| Genetic Information Processing | Replication and repair | 202944.68±34180.09 | 210229.58±40753.89 | 222816.91±37185.10 | 0.648 | 0.532 | 0.758 | 0.911 | 0.508 |
| Cellular Processes | Cell motility | 145489.77±33924.62 | 142350.46±40305.97 | 166532.40±44221.74 | 0.987 | 0.387 | 0.413 | 0.985 | 0.509 |
| Environmental Information Processing | Membrane transport | 143878.62±24813.54 | 146359.61±28036.15 | 138015.65±23583.66 | 0.253 | 0.778 | 0.770 | 0.977 | 0.878 |
| Metabolism | Glycan biosynthesis and metabolism | 133661.36±22895.50 | 132842.88±19963.04 | 125338.36±19465.41 | 0.437 | 0.651 | 0.728 | 0.996 | 0.678 |
| Genetic Information Processing | Folding, sorting and degradation | 125401.59±19153.81 | 128416.70±20605.22 | 136706.02±21462.23 | 0.739 | 0.488 | 0.670 | 0.948 | 0.480 |
| Metabolism | Biosynthesis of other secondary metabolites | 114206.86±19838.40 | 112907.90±20368.66 | 106692.33±23206.68 | 0.323 | 0.727 | 0.809 | 0.991 | 0.735 |
| Genetic Information Processing | Translation | 98762.64±16491.43 | 103611.44±23100.33 | 110734.99±21145.57 | 0.782 | 0.469 | 0.743 | 0.870 | 0.440 |
| Metabolism | Nucleotide metabolism | 72940.35±11589.77 | 76122.10±13951.04 | 79082.45±11880.24 | 0.542 | 0.589 | 0.871 | 0.853 | 0.559 |
| Cellular Processes | Cell growth and death | 49850.32±8749.51 | 50388.51±11078.78 | 53111.06±10192.50 | 0.272 | 0.764 | 0.835 | 0.993 | 0.773 |
| Environmental Information Processing | Signal transduction | 31680.60±5961.92 | 32327.46±6886.73 | 32390.49±6902.64 | 0.032 | 0.969 | 1.000 | 0.976 | 0.972 |
| Genetic Information Processing | Transcription | 25579.63±4596.11 | 25306.57±5572.26 | 32646.46±15361.57 | 1.622 | 0.218 | 0.270 | 0.998 | 0.295 |
| Cellular Processes | Transport and catabolism | 13696.20±2633.72 | 13613.44±2815.19 | 11887.64±5079.06 | 0.692 | 0.51 | 0.587 | 0.999 | 0.558 |
| Cellular Processes | Cellular community - prokaryotes | 12594.14±2718.13 | 12753.30±3348.29 | 13761.95±2719.84 | 0.417 | 0.664 | 0.750 | 0.993 | 0.681 |
| Organismal Systems | Environmental adaptation | 10359.46±2034.87 | 10325.06±2216.63 | 10476.35±1430.35 | 0.015 | 0.985 | 0.985 | 0.999 | 0.991 |
| Human Diseases | Infectious diseases | 8711.2±1423.52 | 8593.63±1544.46 | 8450.72±1884.45 | 0.058 | 0.944 | 0.981 | 0.987 | 0.939 |
| Human Diseases | Neurodegenerative diseases | 7932.94±3554.18 | 2266.12±3567.71 | 1373.89±1381.31 | 12.53 | 0 | 0.806 | 0.002 | 0.000 |
| Organismal Systems | Endocrine system | 3467.89±663.71 | 3425.05±803.06 | 3274.18±479.93 | 0.212 | 0.81 | 0.880 | 0.990 | 0.810 |
| Organismal Systems | Immune system | 1873.78±337.31 | 1789.63±369.59 | 2084.88±676.36 | 0.883 | 0.427 | 0.415 | 0.928 | 0.632 |
| Organismal Systems | Digestive system | 411.23±219.95 | 226.55±221.79 | 138.31±158.34 | 4.27 | 0.026 | 0.630 | 0.150 | 0.022 |
| Human Diseases | Cancers | 0.00±0.00 | 229.25±469.17 | 0.00±0.00 | 2.149 | 0.139 | 0.193 | 0.193 | 1.000 |
| Human Diseases | Cardiovascular diseases | 95.23±61.45 | 44.81±20.57 | 44.77±48.19 | 3.511 | 0.046 | 1.000 | 0.076 | 0.076 |
| Organismal Systems | Sensory system | 34.41±64.56 | 0.65±1.04 | 0.58±1.30 | 2.465 | 0.106 | 1.000 | 0.155 | 0.154 |
| Cellular Processes | Cellular community - eukaryotes | 0.36±1.09 | 0.29±0.52 | 0.04±0.09 | 0.547 | 0.586 | 0.380 | 0.978 | 0.492 |
| Human Diseases | Immune diseases | 8.00±14.19 | 8.97±10.00 | 2.48±2.88 | 1.068 | 0.359 | 0.332 | 0.948 | 0.204 |
| Organismal Systems | Development | 0.57±0.98 | 2.56±2.36 | 11.79±23.25 | 1.768 | 0.192 | 0.033 | 0.024 | 0.990 |
| Environmental Information Processing | Signaling molecules and interaction | 0.47±0.94 | 4.25±4.51 | 0.65±1.71 | 5.078 | 0.014 | 0.723 | 0.973 | 0.586 |
| Organismal Systems | Excretory system | 0.03±0.09 | 0.04±0.11 | 0.00±0.01 | 0.447 | 0.645 | 0.668 | 0.995 | 0.728 |

**Supplementary Table S12. Longitude and latitude of sampling sites**

| **Sampling site** | **Longitude** | **Latitude** |
| --- | --- | --- |
| Urban-farmland ecosystem | 36.15512 | 117.1573 |
| Forest-urban ecosystem | 36.19347 | 117.1198 |
| Forest ecosystem | 36.27721 | 117.0628 |

**References**

Ambika Manirajan B, Ratering S, Rusch V, Schwiertz A, Geissler-Plaum R, Cardinale M, et al. Bacterial microbiota associated with flower pollen is influenced by pollination type, and shows a high degree of diversity and species-specificity. Environ Microbiol 2016; 18: 5161-5174.

Anderson KE, Sheehan TH, Mott BM, Maes P, Snyder L, Schwan MR, et al. Microbial Ecology of the Hive and Pollination Landscape: Bacterial Associates from Floral Nectar, the Alimentary Tract and Stored Food of Honey Bees (Apis mellifera). PLOS ONE 2013; 8: e83125.

Biao T, Yi L, Rui L, Xiunan Z, Ling Z, Mingrong Q, et al. Analysis of Microbial Contamination and Community Structure of Bee Pollen. Food Science 2020; 41: 325-331.

Disayathanoowat T, Li H, Supapimon N, Suwannarach N, Lumyong S, Chantawannakul P, et al. Different Dynamics of Bacterial and Fungal Communities in Hive-Stored Bee Bread and Their Possible Roles: A Case Study from Two Commercial Honey Bees in China. Microorganisms 2020; 8: 264.

Wang Y, Li Z, Ma L, Li G, Han K, Liu Z, et al. The Native Dietary Habits of the Two Sympatric Bee Species and Their Effects on Shaping Midgut Microorganisms. Frontiers in Microbiology 2021; 12: 738226.
